# Exploring the therapeutic potential of cannabidiol in soft tissue wound healing: Delivery strategies and anti-inflammatory pathways

**DOI:** 10.1016/j.apsb.2025.10.001

**Published:** 2025-10-10

**Authors:** Arita Dubnika, Inga Jurgelane, Andra Grava-Ceplite, Selay Tornaci, Natalia N. Porfiryeva, Diana Solovyov, Nabanita Saha, Nibedita Saha, Elina Kelle, Dagnija Loca, Ebru Toksoy Öner, Alejandro Sosnik

**Affiliations:** a Institute of Biomaterials and Bioengineering, Faculty of Natural Sciences and Technology, Riga Technical University, Riga LV-1048, Latvia; b Baltic Biomaterials Centre of Excellence, Headquarters at Riga Technical University, Riga LV-1048, Latvia; c IBSB, Department of Bioengineering, Marmara University, Istanbul 34854, Turkey; d Laboratory of Pharmaceutical Nanomaterials Science, Department of Materials Science and Engineering, Technion-Israel Institute of Technology, Haifa 3200003, Israel; e Centre of Polymer Systems, University Institute, Tomas Bata University, Zlin 76001, Czech Republic

**Keywords:** Medical *cannabis*, Cannabidiol, Drug delivery, Soft tissue wound healing, Soft tissue inflammation, Drug formulations, Cannabidiol administration route, Cannabidiol clinical trials

## Abstract

This review explores the molecular and cellular pathways of soft tissue wound healing and the potential therapeutic use of the non-psychotropic cannabinoid cannabidiol (CBD), integrating findings from *in vitro* and *in vivo* preclinical studies as well as completed and ongoing clinical trials. It provides a comprehensive summary of the next steps in new CBD-based product development by analyzing current trends in dosage optimization, treatment guidance, delivery systems, ranging from liposomes, microemulsions to hydrogels. Additionally, the review examines clinical trials related to CBD formulations, delivery routes, and participant outcomes, offering a deeper understanding of the mechanisms guiding the activity beyond binding to cannabinoid 1 (CB1) and CB2 receptors. Furthermore, it highlights challenges and future perspectives in CBD formulation studies, presenting both currently studied approaches and emerging possibilities for innovation. Therapeutic potential of CBD has proved itself in the recent years and only regulatory issues and clarity in treatment and delivery routes will limit its widespread use in soft tissue healing.

## Graphical abstract

Rapidly expanding clinical trials are advancing cannabidiol delivery systems for diverse soft tissue wound healing applications, emphasizing varied administration forms and routes to enhance repair, regeneration, and recovery outcomes.

**Figure:**
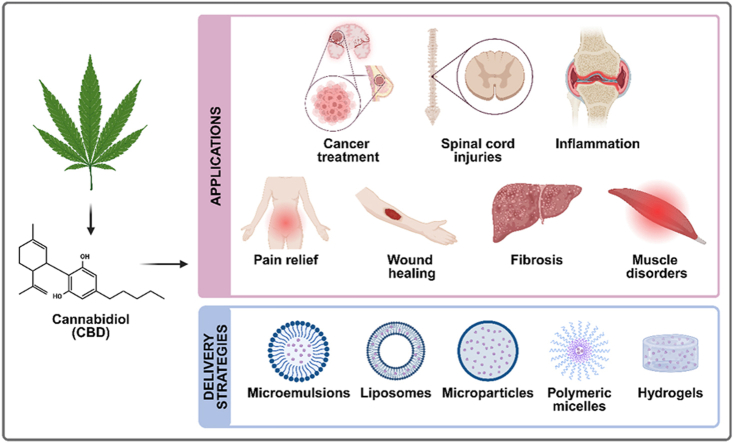

## 1 Introduction

**Figure 1:**
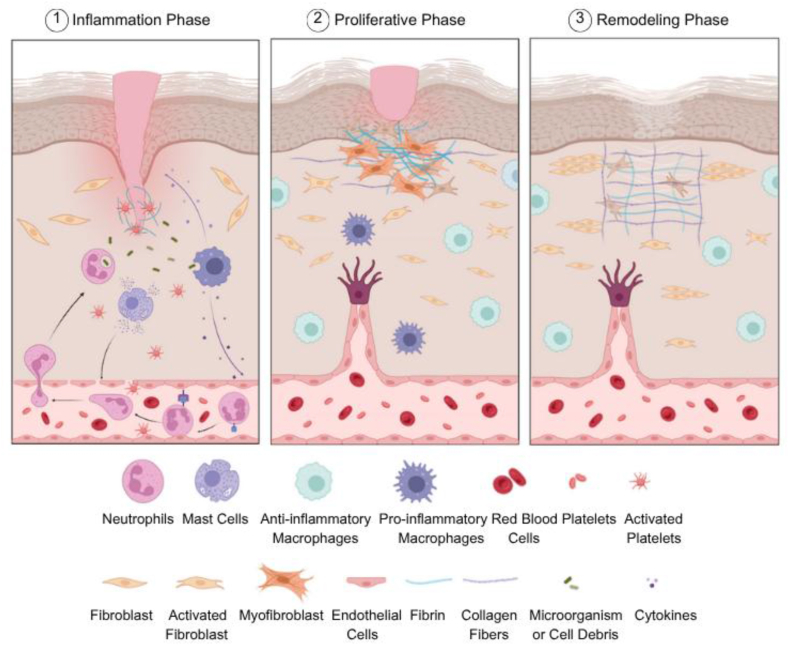
Mechanisms of wound healing. Figure created with BioRender.

Soft tissue is the supporting, extra-skeletal tissue of organs other than epithelial tissue, which includes connective and adipose tissues, skeletal muscle, tendons, ligaments, blood and lymph arteries, peripheral nerves, and other fibrous tissues that cover most of the body^1^. If these tissues are disrupted due to various causes, such as physical, mechanical or chemical injuries or pathological conditions, a well-organized three-stage wound healing process is initiated (Fig. 1)^2^. This process begins with the inflammation phase, during which dead cell debris and invading microorganisms are removed. It is followed by the proliferative phase, characterized by re-epithelialization and tissue granulation, and concludes with the remodeling phase^3^^,^^4^. The healing process involves a variety of cell types, including endothelial cells, keratinocytes, fibroblasts, and immune cells. These cells have distinct roles in the process by secreting mediators such as growth factors, cytokines, interleukins, interferons, and matrix metalloproteinases (MMPs). While growth factors encourage the proliferation of diverse cell populations, cytokines recruit the appropriate cell types to the wound site and control the immune response. Additionally, zinc-dependent MMPs play an important role in cell migration and extracellular matrix (ECM) remodeling^5^, ^6^, ^7^, ^8^.

Biological function disorders, including diabetes, age, obesity, and other pre-existing medical conditions, can interfere with and impair the wound healing process. Bioactive substances that influence the behavior, activation, and recruitment of the cells involved in wound healing are employed to accelerate or ensure the effective progression of this intricate process^9^. Active substances with antioxidant and anti-inflammatory properties play a crucial role in the treatment of skin and soft tissue infections, which are often associated with soft tissue damage^10^. Natural therapeutic agents such as polyphenols can be extracted from plants (*e.g*., Aloe vera) or marine organisms and exhibit remarkable properties, including antimicrobial, regenerative and antioxidant activities^11^^,^^12^. Curcumin, resveratrol, and tannic acid are among the well-known polyphenolic compounds that contribute to accelerating the soft tissue wound healing process^13^. Another example of an active substance is cannabidiol (CBD), which has demonstrated excellent antioxidant, anti-inflammatory^14^, and neuromodulator^15^ properties.

The therapeutic properties of *Cannabis sativa* have been recognized for over five millennia, with it use as a medical substance dating back to the Christian era in Central and Northeast Asia, particularly in India^16^, ^17^, ^18^. However, despite its potential, this plant has undergone limited scientific exploration of its chemical and biological properties, and its medical use has remained restricted, particularly following its classification as a substance with no medical value under the United Nations Single Convention on Narcotic Drugs^19^. Only in 1964, Gaoni and Mechoulam, researchers at the Hebrew University of Jerusalem described the isolation and the chemical structure of the psychoactive Δ^1^-3,4-*trans*-tetrahydrocannabinol for the first time^20^. Cannabinoids can be extracted naturally from the *Cannabis sativa* plant, gained by isomerization of CBD, or synthesized^21^. To date, over 100 phytocannabinoids (namely isolated from the *Cannabis* plant) of 11 different chemical families have been identified, including (−)-Δ^9^-tetrahydrocannabinol (THC), CBD, cannabigerol (CBG), cannabichromene (CBC), cannabinodiol (CBND), cannabielsoin (CBE), cannabicyclol (CBL), cannabinol (CBN), cannabitriol (CBT), and over 100 terpenes^22^.

In the 1980s, Allyn C. Howlett and coworkers at the St. Louis University Medical School discovered the cannabinoid receptor 1 (CB1), which is targeted by Δ^9^-THC in the central nervous system (CNS)^23^, ^24^, ^25^. In the following years cannabinoid receptor 2 (CB2) was identified. It is the peripheral receptor of cannabinoids and it has been shown to modulate the immune system and mediate anti-inflammatory responses^26^, ^27^, ^28^. Upon the elucidation of diverse molecular targets and signaling pathways of cannabinoids in the CNS and periphery^25^^,^^29^, natural and synthetic cannabinoids have been proposed as therapeutic agents in the treatment of local and systemic diseases and some of them are approved by the regulatory agencies^30^, ^31^, ^32^, ^33^.

Unlike THC, CBD is a non-psychoactive compound isolated from the *Cannabis sativa* plant. It has been described to have important medical properties such as antibacterial, anticancer, anti-inflammatory, antioxidant, antiepileptic, anti-depressive and neuroprotective^34^. For example, CBD has been proposed in the treatment of neurodegenerative and inflammatory diseases (*e.g*., inflammatory bowel diseases, arthritis, psoriasis, periodontitis), as an anti-convulsant in epilepsy, and the amelioration of the symptoms of autism spectrum disorders, post-traumatic stress disorders, multiple sclerosis, depression, anxiety disorders, chronic pain, among other conditions^14^^,^^19^^,^^35^, ^36^, ^37^, ^38^, ^39^, ^40^, ^41^, ^42^, ^43^, ^44^, ^45^, ^46^. Furthermore, cannabinoids have been found to induce stem cell differentiation and they have been incipiently investigated in tissue engineering and regenerative medicine^47^^,^^48^.

The most common administration strategies for *Cannabis* extracts and its main active compounds, namely the psychoactive THC and the non-psychoactive CBD, are the oral route (*e.g*., oils, capsules and pills) and inhalation (*e.g*., smoking and vaping). A major drawback of the oral route is the relatively low bioavailability of cannabinoids (less than 19%) mainly owing to their limited aqueous solubility and hepatic first-pass metabolism^49^, ^50^, ^51^. Several countries have authorized the inhalation of medicinal cannabinoids^52^ and, even if it results in higher systemic bioavailability (up to 35%–50%) than the oral route, it is intimately associated with cardiovascular risks and severe adverse respiratory effects^53^. To overcome these issues, the use of aerosolizers and vaporizers has been proposed^54^. According to a survey by Spinella et al.^55^, which explored people's beliefs about the effects of CBD, THC, and their combinations, it was noted that participants generally had greater expectations regarding products containing CBD compared to those containing THC. These results can be explained by the growing interest in CBD products in research and development and ongoing clinical trials due to its non-psychotropic activity, as opposed to THC. A recent study has shown that younger individuals tend to favor novel administration methods for CBD, such as vaping or drinking, followed by sublingual and oral administration routes, indicating that they are more open to testing alternative administration routes and advanced delivery systems that result higher bioavailability^56^. However, ingestion and inhalation are appropriate for systemic treatments and are associated with off-target toxicity, while they are less convenient for local ones such as soft tissue inflammation and wound healing, for which alternative administration approaches and delivery systems must be developed.

The increasing legalization and widespread use of cannabis products worldwide are driving the rapid growth of the market for different *cannabis*-based products. The global market of medicinal *Cannabis* has been valued at $21.0 billion in 2023 and is expected to expand at a compound annual growth rate of ∼25% to reach $149 billion by 2031^57^^,^^58^. At the same time, the heterogeneity and complexity, and the inter-batch variability of *Cannabis* extracts pose significant challenges to their translation into pharmaceutical products^59^. Furthermore, the poor aqueous solubility of many cannabinoids makes the development of pharmaceutical formulations difficult and results in low oral bioavailability^60^^,^^61^. The aqueous solubility of CBD is ∼0.01 mg/mL^62^, being classified as Class II of the Biopharmaceutical Classification System (low aqueous solubility and high permeability), and exhibiting an oral bioavailability <20%^50^^,^^63^. Thus, there is a growing need to design and develop biomaterial-mediated cannabinoid delivery strategies to overcome inherent biopharmaceutical limitations, exert precise spatiotemporal control over release kinetics, and thereby facilitate the translation of CBD-based products into the clinics.

Investigations on CBD and cannabis plant, in general, are active areas of multidisciplinary research, involving engineers, chemists, physicists, biologists, and medical experts. Additionally, the implementation of cannabinoid-based solutions affects and involves a wide range of stakeholders, including healthcare delivery organizations, health plans and employers, private research institutes, government authorities, public service boards, research institutes, associations, and academics. Their use has the potential to improve the healthcare system by enabling new efficiencies and lowering costs while creating value for patients and increasing satisfaction^64^^,^^65^.

Current understanding of the molecular pathways of CBD in soft tissue healing is limited. Mechanisms of action remain unclear, and clinical evidence is lacking, hindering dosage optimization and treatment guidance. Product quality and variability, as well as regulatory discrepancies, pose additional challenges. Robust research, clinical trials, and regulatory clarity are crucial to elucidate its therapeutic potential, determine optimal dosing, and ensure its safe and effective utilization in soft tissue healing.

The investigation of CBD in wound healing and inflammation and the development of delivery systems has been extensively reviewed in the literature^65^, ^66^, ^67^. However, these works do not emphasize the research at the interface of the therapeutic use of CBD in soft tissue wound healing and local delivery systems, and the challenges faced to optimize the therapeutic outcomes. In this framework and given the growing interest and scientific inquiry into the therapeutic potential of CBD, this review rigorously overviews the applications of CBD in soft tissue wound healing, the delivery systems developed to ensure local release and reduce systemic exposure and off-target toxicity and discusses the possible anti-inflammatory pathways involved in the healing process. Furthermore, relevant clinical data are included to substantiate and guide future research directions. Overall, this work contributes to evidence-based decision-making and provides direction for future research endeavors in this promising area of study.

## 2 CBD in soft tissue healing

CBD has long been recognized for its influence on critical healing processes, including immune system response, metastasis, and cell proliferation, though its mechanisms of action are still under investigation. A recent study has demonstrated that mesenchymal stromal cells treated with lipopolysaccharides and subsequently exposed to CBD experienced reduced oxidative stress and overcame the inhibition of adipogenic and chondrogenic differentiation^68^. Similarly, research by Atalay et al.^69^ revealed that CBD decreased lipid peroxidation in keratinocytes exposed to hydrogen peroxide and prevented protein aggregation by preserving chaperone functionality. Further studies explained that CBD is effective against oxidative stress *via* increasing superoxide dismutase (SOD) and decreasing the expression of nicotinamide adenine dinucleotide phosphate oxidase (NOX) genes, which are involved in the production of reactive oxygen species (ROS) during tissue healing^70^.

Another mechanism of action of CBD is its neuromodulatory effect on the CNS^15^^,^^71^. The endocannabinoid system (ECS) plays a crucial role in neural modulation and maintaining homeostasis. The system includes cannabinoid receptors CB1 and CB2 and protein transporters, which help to interact with other signaling pathways. CBD is also a ligand for cannabinoid receptors, therefore attracting great attention as a therapeutic agent^72^. A recent study on mice showed that CB1 and CB2 receptors have an essential role in regulating inflammation during wound healing, and there is a delay in wound healing, especially in CB1-deficient mice. For this reason, cannabinoids, which are substrates of CB1 and CB2, have essential effects on tissue healing^73^. Further studies on inflammation and CBD have shown that the strong anti-inflammatory properties of CBD help to prevent the formation of abnormalities that occur during soft tissue healing, such as fibrosis^74^. In their study on fibroblast and keratinocyte cells, Sangiovanni et al.^75^ reported that CBD inhibited nuclear factor kappa B (NF-*κ*B)-mediated transcription induced by tumor necrosis factor-*α* (TNF-*α*), which plays an important role in skin inflammation. In another study, it was observed that the addition of CBD nanoemulsions combined with lipopolysaccharides to monocyte cells resulted in decreased expression of TNF-*α* and the proinflammatory interleukin (IL)-6. This inhibition was attributed to the suppression of CB2 receptor activity or the inhibition of NF-*κ*B-mediated transcription. Additionally, the same study reported high wound closure rates in human corneal epithelial cells, which was attributed to enhanced proliferation and migration stimulated by epithelial growth factor through CB1 receptor activation^76^. Furthermore, extensive evidence in the literature has highlighted the ability of cannabinoids to influence immune cell behavior, including the regulation of proliferation, apoptosis, and cytokine production, such as IL-1*β*, IL-6, IL-4, IL-5, IL-12, TNF-*α*, and other^77^.

Soft tissue wound healing is a vital and tightly regulated biological process essential for survival^78^. The primary objective of wound healing is tissue repair and wound closure, often without consideration for scar formation, which typically results from excessive fibroblast proliferation, myofibroblast activation, and overproduction of ECM components. The formation of scar tissue may not be eliminated during the remodeling phase due to clinical complications such as diabetes or venous insufficiency and may lead to the formation of fibrosis or chronic wounds due to an unsuppressed immune response^79^. Achieving scarless healing has become a major therapeutic goal, aiming to prevent fibrosis and preserve the tissue's original architecture and functionality during the healing process.

In recent years there have been different approaches to promote scarless healing, such as using biomaterials like nanofibers^80^ or hydrogels^81^ or biomedical technologies such as cell therapy or drug therapy^79^. CBD may be a helpful bioactive supplement especially with its TGF-*β*1 inhibitory effect and triggering M2 polarization from M1 macrophages *via* the CB2 receptor and generally reducing inflammation. In addition, preclinical studies have reported that CBD triggers the activation of progenitor or stem cells, reduces oxidative stress, and can modulate MMPs and tissue inhibitors of metalloproteinases (TIMPs) which are key enzymes of ECM remodeling^66^^,^^82^^,^^83^. These features made CBD a promising candidate in enhancing soft tissue repair, particularly in challenging scenarios of chronic wounds like diabetic wounds. Due to diabetes, accumulation of ROS, hypoxia caused by vascularization disruption and immune system dysregulation leads to patient develop non-healing chronic diabetic wounds^84^.

To date, there is no gold standard therapy for diabetic wounds; however, current research focuses on approaches such as regulation of the MMPs, growth factors, and immune-centered therapies. Given its bioactive properties, CBD is predicted to be a promising candidate for diabetic wound treatment. In a recent study by Shah et al.^85^ wound models were established in both wild-type (C57BL/6) and diabetic (*db/db*) mice, and CBD was applied topically or *via* inhalation. The results showed that in diabetic mice, CBD treatment nearly doubled the expression of connective tissue growth factor (CTGF), which stimulates fibroblast proliferation, promotes their differentiation into myofibroblasts, and triggers angiogenesis through endothelial cell proliferation. One of the major challenges in diabetic wound healing is infection and microbial colonization, which lead to persistent inflammation and continuous immune activation. This chronic immune response can damage newly formed tissue and severely impair proper healing^86^.

The wound microbiota plays a crucial role in the healing of chronic wounds. Recent studies have shown that CBD may positively influence the wound microbiome. For instance, CBD and CBG have demonstrated the ability to inhibit biofilm formation by *Pseudomonas aeruginosa* and *Escherichia coli*, while exhibiting minimal impact on *Staphylococcus epidermidis*—a common and typically beneficial component of healthy skin microbiota. Notably, both cannabinoids showed stronger inhibitory effects against *Staphylococcus aureus*, a known skin pathogen^87^. In another recent study, Yin et al.^88^ developed CBD-loaded, chitosan-modified silver nanoparticles designed to combat polymicrobial infections. These nanoparticles enabled the controlled release of CBD, effectively prevented biofilm formation by methicillin-resistant *S. aureus* and *E. coli*, reduced oxidative stress, and promoted vein endothelial cell proliferation—an important factor for vascular regeneration and wound healing^87^. Additionally, a recently compiled systematic review reported that CBD has beneficial effects on gut microbiota, reduces human immunodeficiency virus (HIV)-associated intestinal inflammation, enhances immune function, and contributes to pain management^33^.

Although CBD has a wide range of applications, most research has been focused on its use as pain killer and to alleviate side effects of chemotherapy such as nausea and dizziness, and as antimicrobial agent^89^. More recently, its use in dentistry in general and in the healing of soft buccal tissues in particular has been reviewed^70^.

## 3 CBD mechanisms of action and potential applications in soft tissue conditions

### 3.1 Pain relief before and after surgery

Neuropathic pain is defined as pain resulting from damage or disease affecting the somatosensory system^90^. It can arise from various causes, including metabolic disorders, viral infections, autoimmune disorders, chemotherapy-induced neuropathies, traumatic damage to the nervous system, inflammatory disorders, channelopathies and hereditary neuropathies^91^. As surgeons are becoming more and more interested in the use of CBD to relieve patient pain^92^, extensive research studies have investigated the use of opioids for pain relief after surgeries. Moreover, there are studies that propose that CBD could replace even morphine^92^^,^^93^.

CBD influences pain management by targeting ion channels of the transient receptor potential (TRP) family (TRPV1, TRPA1 and TPRM8) and glycine receptor (GlyR), non-cannabinoid G protein-coupled receptors (GPCRs) and peroxisome proliferator-activated receptors (PPARs), by inhibiting *N*-arachidonylethanolamine (AEA) and frailly slowing down the drug hydrolysis by enzyme fatty acid amide hydrolase (FAAH)^94^. Furthermore, CBD enhances the targeting of CB1 and CB2 receptors by increasing AEA levels. CB1 receptors are associated with signaling to the brain and CNS, while CB2 receptors are primarily linked to the immune system and peripheral nervous system^95^^,^^96^.

**Figure 2:**
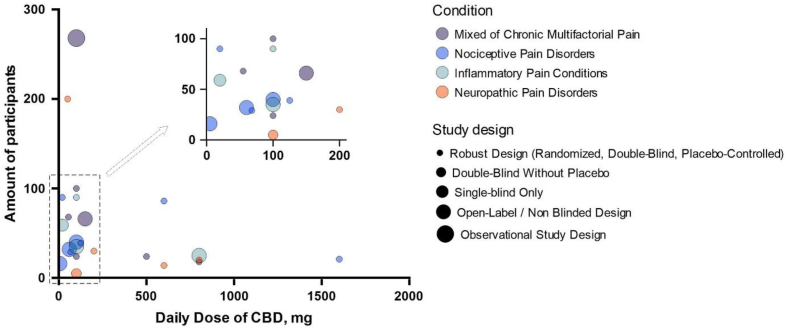
Comparative analysis of key variables across clinical studies on CBD targeting soft tissue pain management.

According to the Clinical Trials data base (https://clinicaltrials.gov/) there are currently a total of 484 studies that include the keywords “cannabidiol” and “CBD”, from which 204 are completed (till May 22, 2025). Clinical trials specifically targeting soft tissues are summarized in Table 1. These clinical trials show promising but variable results due to differences in study design, dosage and administration forms (tablets, capsules, gels, and oily solutions for oral, buccal, and topical administration). Fifteen of the indicated studies have robust study design (randomized, double-blind and placebo controlled), with low design limitation risk. While eight studies are open-label, non-blinded, observational or single blinded only design, limiting the strength of their conclusions, due to the high risk of performance and detection bias and no intervention control. Most studies are based on the oral delivery, which is associated with poor bioavailability and inconsistent absorption. Alternative delivery routes like sublingual, topical, and rectal are rare, and no trials to date have tested an encapsulated or controlled-release CBD formulation. According to the specified clinical trials, CBD is most commonly used for neurological conditions and far less frequently for soft tissue pain relief. Comparative analysis of the key variables across clinical studies is summarized in Fig. 2. The main reason for this limited therapeutic scope may be largely attributed to regulatory restrictions and CBD's biopharmaceutical challenges, such as poor water solubility and high chemical instability, which further complicate the development of new products and their market distribution.

**Table 1:** Clinical trials with CBD targeting soft tissue pain management.

Identification No. and design limitations	Condition	Number of participants	Intervention	CBD administration form	Trial location
NCT03233633	Pain	66	Medical marijuana (high CBD:THC ratio) thrice daily for 5 days	Oral capsule	United States
Open-label					
Non-randomized					
NCT04059978	Acute nociceptive pain, Hyperalgesia, Allodynia opioid-induced hyperalgesia	21	CBD, 1600 mg single dose and remifentanil 0.1 μg/kg/min i.v. for 30 min	Oral solution	Switzerland
Randomized					
Double-blind					
Placebo-controlled					
NCT04607603	Osteo arthritis, knee pain, joint	86	Cannabidiol, 200 mg thrice daily for 7 weeks	Oral capsule	Austria
Randomized					
Double-blind					
Placebo-controlled					
NCT05498012	Chronic periodontitis	90	CBD 1% (*w*/*w*) gel, applied by dentist after oral hygiene, CBD 1% (*w*/*w*) toothpaste for daily use by patient	Dental gel, toothpaste	Czech Republic
Randomized					
Double-blind					
Placebo-controlled					
NCT04193631	Musculoskeletal pain	16	CBD, 5 mg single dose when patients experience pain	Sublingual tablet	United States
Open label					
Non-randomized					
NCT05494788	Recurrent pericarditis	25	CardiolRx (CBD), twice daily for 8 weeks, initial dose 5 mg/kg of body weight, from day 3 p.m. 7.5 mg/kg of body weight, from day 10 p.m. 10.0 mg/kg of body weight	Oral	United States
Open-label					
Non-randomized					
NCT03522324	Chronic pain, chronic Low back pain	268	Cannabis edible, edible product of choice, used as necessary	Oral	United States
Observational					
Non-randomized					
NCT04760613	Radiculopathy	14	CBD, 600 mg, daily use	Oral capsule	United States
Randomized					
Double-blind					
Placebo-controlled					
NCT04088929	Diabetic neuropathies	32	CBD, 20 mg trice daily for three weeks	Sublingual tablet	United States
Open-label					
Non-randomized					
NCT02751359	Pain	18	CBD, 200, 400 or 800 mg daily for three days	Oral	United States
Randomized					
Double-blind					
Placebo-controlled					
NCT04754399	Arthralgia, breast cancer	40	CBD twice daily, 25 mg (week 1), 50 mg (week 2), 75 mg (week 3), 100 mg (week 4+) for 15 weeks	Oral solution	United States
Open-label					
Non-randomized					
NCT04271917	Pain, acute	68	CBD oil (17 or 37 mg/mL) 0.5 mL thrice daily for 5 days	Oil placed under the tongue for 30 s then swallowed	United States
Randomized					
Double-blind					
Placebo-controlled					
NCT04298554	TMJ disorder, Myofacial pain, TMD	59	CBD oil (20 mg/mL) 1 mL daily	Oil placed under the tongue for 1 min then swallowed	United States
Single-blind					
Non-randomized					
NCT04672252	Pain, Postoperative	100	CBD administrated with routine post-operative pain management regimen	Oral disintegrating tablet	United States
Randomized					
Double-blind					
Placebo-controlled					
NCT04586712	Muscle injury, pain relief	29	Active CBD-extract (2000 mg/30 mL hemp extract) 25 mg/day or 67.5 mg/day	Oral liquid	United States
Randomized					
Double-blind					
Placebo-controlled					
NCT04387617	Urinary Stone, pain, Postoperative	90	CBD oil, 20 mg daily for three days	Oral liquid	United States
Randomized					
Double-blind					
Placebo-controlled					
NCT04226690	Pain	24	Medical marijuana medicine (CBD (40 or 100 mg) or CBD/THC (40/10, 40/20 or 100/30 mg)) for 7 days	Oral tablet	United States
Randomized double-blind					
Placebo-controlled					
NCT03985995	Pain sensation, Hyperalgesia, Allodynia	20	CBD (100 mg/mL) single dose of 800 mg daily	Oral solution	Switzerland
Randomized double-blind					
Placebo-controlled					
NCT03099005	HIV neuropathy, pain syndrome	5	THC/CBD (1.9% THC + 0.01% CBD or 1.9% THC + 0.01% CBD or 1.4% THC + 5.1% CBD)	Vaporized cannabis	United States
Randomized					
Double-blind					
NCT05388058	Chemotherapy-induced peripheral neuropathy, malignant solid neoplasm	30	CBD cream twice per day for 14 days	Topical cream	United States
Randomized double-blind					
Placebo-controlled					
NCT04729179	Fibromyalgia	200	CBD, 10 mg/day initial dose, dose escalation every third day until the maximum dose 50 mg/day is reached (in two weeks), dose 50 mg/day is taken for 24 weeks	Oral tablets	Denmark
Randomized double-blind					
Placebo-controlled					
NCT04044729	Chronic pain	24	500 mg of CBD suspended in medical grade olive oil and mixed into chocolate pudding, one a day for 5 days	Olive oil	United States
Randomized					
Double-blind, placebo-controlled					
NCT06968910	Attenuate non-bacterial prostatitis symptoms	35	CANNEFF® SUP suppositories with CBD (100 mg) and hyaluronic acid (6.6 mg), each night for 30 days	Rectal suppositories	Czech Republic
Open-label					
Non-randomized					
NCT03825965	Persistent post-surgical pain following TKA	39	Oral formulation CBD:THC (50:2 mg/mL), 125 mg CBD/5 mg THC daily for 6 months	Oral oil solution	Canada
Randomized					
Double-blind, placebo-controlled

### 3.2 Fibrosis

Fibrosis is a pathological condition characterized by excessive growth, stiffness, and sometimes scarring of different tissues or organs due to the replacement of parenchyma with connective tissue during the healing process, resulting in an overaccumulation of ECM components and among them, especially collagen^97^. It results from repetitive stimulation of various immunological and molecular mechanisms involved in the healing process after tissue injuries. These injuries may be caused by chronic inflammatory disorders or other types of major tissue injuries^98^. In fact, fibrosis can affect many organs such as the heart, liver and lungs and in severe cases it can lead to death^99^.

Wound healing is a complex process that starts with coagulation and inflammation and continues with tissue formation and remodeling. When tissue damage occurs, pro-inflammatory cytokines such as IL-1, IL-6, and TNF are secreted from the damaged tissue to induce macrophages, while thymic stromal lymphopoietin (TSLP), IL-25, IL-33, and IL-5 are released to stimulate eosinophils. This activation of the innate immune system, when maintained in a controlled manner, promotes the transition of fibroblasts into myofibroblasts, ultimately triggering tissue formation^98^. The control of the acute immune response is provided by cytokines such as IL-10 released by T regulatory (Treg) cells^100^. During tissue formation, fibroblasts are attracted to the wound area, and ECM components such as procollagen, elastin, proteoglycans, and hyaluronic acid (HA) are produced to form new tissue structures in the wound area. Until the ECM remodeling stage, the final stage of wound healing, the ECM is produced in a dispersed and unorganized structure^101^. MMPs and TIMPs are enzymes that control cell adhesion and regulate the amount of collagen expression in fibroblast cells, especially during the remodeling phase, where defects in this regulation can lead to collagen deposition due to fibroblasts producing collagen faster than it can be degraded^77^^,^^102^. When the damage is major or repetitive, like in chronic inflammation, ECM components such as collagen and fibronectin disproportionately accumulate, resulting in fibrosis^103^. The accumulation of ECM components during fibrosis causes the tissue to harden and lose its flexibility, resulting in a reduction of the functionality of the affected organs. The basic mechanism in the formation of fibrosis can be examined under two headings. The first one is the case of an unregulated immunological response. During wound healing, platelets and fibrin clump and create fibrin clots, which in turn cause the secretion of transforming growth factor beta-1 (TGF-*β*1) from platelets for the differentiation of fibroblasts to myofibroblasts producing ECM proteins. Any regulation problem in this system may lead to fibrosis^104^. Furthermore, neutrophils and macrophages are needed to clean cellular debris and bacteria from the wound area, however, if these cells and the mediators such as ROS they secrete are not removed from the environment quickly, the inflammatory response continues to increase and damage the surrounding healthy tissue repeatedly which may result in fibrosis^103^. It has been shown that cytokines such as TNF-*α*, IL-1*β* and IL-6 can also cause dose-dependent fibrosis^105^^,^^106^. Adaptive immune cells are effective in the formation of fibrosis. T helper 17 (Th17) cells are known to secrete IL-17 and it has been reported that IL-17 increases the activity of neutrophils that cause fibrosis by triggering cell apoptosis in the tissue. Moreover, it is known that there is an interaction between IL-17 and TGF-*β*1^103^^,^^107^. It has been reported that T helper 2 (Th2) cells show organ-specific pro-fibrotic effects by secreting IL-13 and IL-4^107^^,^^108^. However, studies have also shown that there is a negative regulation with the help of antifibrotic cytokines such as interferon (IFN)-*γ* secreted by T helper 1 (Th1) cells, yet the regulation is still unclear^109^^,^^110^. Another issue that may cause fibrosis is cellular-based problems, such as epigenetic changes in some cells. Uncontrolled transformation of fibroblast cells into myofibroblasts, endoplasmic reticulum stress, and telomere shortening in mesenchymal stem cells are among the other factors causing fibrosis^103^.

Although the mechanism of fibrosis has not been entirely resolved, treatment or prevention is aimed at using known pathways and specific proteins such as MMPs, growth factors and TNF-*α*. However, no solution has provided complete recovery from fibrosis yet. Therefore, molecules with anti-inflammatory, anti-oxidative, and anti-fibrotic effects are frequently tested for the prevention and treatment of fibrosis^98^. Anti-inflammatory approaches are based on small molecules that can easily enter the intracellular area and the main targets are nuclear receptors, enzymes, or microRNAs (miRNAs). There is also a multi-component therapy which is the combination of different drugs (such as Fuzheng Huayu capsule for liver fibrosis, Qishenyiqi for ischemic heart fibrosis, and Dahuang Zhechong pill for liver fibrosis) interacting with different pathways such as transforming growth factor beta (TGF-*β*)/MMP-2, TNF/TGF-*β*/*β*-catenin, alpha smooth muscle actin (*α*-SMA)/TNF-*α*/IL-13/p38, and mitogen-activated protein kinase (MAPK)/extracellular signal-regulated kinase (ERK), causing fibrosis simultaneously^111^. However, there are only three U.S. Food and Drug Administration (FDA)-approved medications, namely nintedanib (for pulmonary fibrosis), pirfenidone (for idiopathic pulmonary fibrosis) and ruxolitinib (for myelofibrosis), that are effective only in specific types of fibrosis^77^. Nevertheless, the development of novel and effective pharmacological treatments is required to mitigate or halt the progression of fibrosis, and nature may be a good source for this research. Bioactive compounds, such as polyphenols like resveratrol, curcumin, and ellagic acid, have been shown potential as therapeutic agents for fibrosis due to their abilities such as anti-inflammatory, antioxidant, antibacterial, and more. These substances can regulate fibrogenic processes by either decreasing the formation of ECM proteins implicated in fibroblast-to-myofibroblast differentiation or increasing their breakdown^112^.

**Figure 3:**
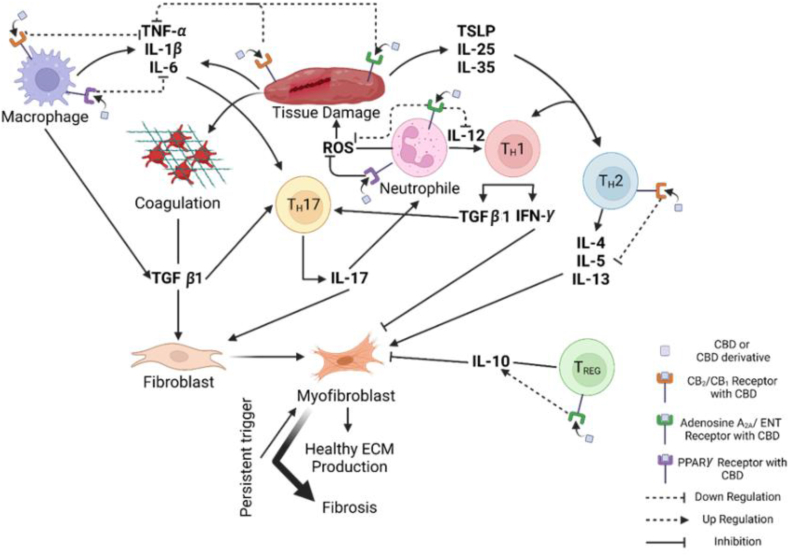
Mechanisms involved in CBD inactivated fibrosis. Figure created with BioRender.

CBD and its derivatives are also highlighted in the literature as preventive or therapeutic agents for various types of fibrosis. For instance, Aljobaily et al.^113^ observed a reduction of hepatic fibrosis in the non-alcoholic steatohepatitis mice model when CBG (a cannabinoid that binds CB1 and CB2 receptors) was delivered through intraperitoneal injections. This study also revealed that TGF-*β*1 secretion from mast cells was reduced which suppresses immune response and helps to reduce hepatic fibrosis. In a similar study by Vuolo et al.^114^ it was reported that intraperitoneal injection of CBD exhibited antifibrotic effect mediated through CB1 and CB2 receptors in allergic asthma-induced BALB/c mice. If the CBD would be encapsulated in a targeted carrier, the reduction of airway inflammation and fibrosis could be decreased more effectively through increased bioavailability to lung tissues and sustaining therapeutic levels. This would likely enhance the suppression of cytokines and further decrease collagen deposition compared to unencapsulated CBD. Overall, CBD may aid in the treatment of fibrosis by interacting with various pathways that promote anti-inflammatory and antioxidant activities (Fig. 3). As an example, CB1 and CB2 are promising targets for fibrosis treatment due to their expression in various immune cells, including natural killer cells, macrophages, and T and B cells. These cells are involved in processes like ROS formation and TNF-*α* production. The interaction of CBD and its derivatives with these receptors elicits both anti-inflammatory and antioxidant effects^14^. Additionally, CBD may aid in fibrosis treatment by acting as a ligand for adenosine A2A receptors. The binding of extracellular adenosine to these receptors deactivates immunosuppressive mechanisms, but CBD competitively binds to the receptors, preventing this deactivation^34^. Furthermore, CB2 plays a critical role in immune modulation, and CB2 agonists have been shown to regulate Th17/Treg polarization^115^^,^^116^ by activating signal transducer and activator of transcription 5 (STAT5) and c-Jun N-terminal kinases (JNK1/2) pathways, thereby reducing inflammatory responses. Naive CD4^+^ T cells differentiate into Th17 cells—which promote inflammation—in the presence of proinflammatory cytokines such as IL-6 and IL-21. In contrast, in the absence of these cytokines, naive CD4^+^ T cells differentiate into Treg cells, which release anti-inflammatory cytokines such as IL-10 and TGF-*β*^117^. A recent study by Tan et al.^118^ demonstrated that phytocannabinoids (*e.g*., CBD and CBG) and terpenes derived from *Cannabis sativa* suppress proinflammatory cytokine secretion in CD3/CD28 and lipopolysaccharide-activated peripheral blood mononuclear cells. This immunomodulatory effect may help mitigate the repetitive immune activation that contributes to fibrosis. It is reported in the literature that CBD and its derivatives can also interact with PPAR gamma (PPAR-*γ*). Activation of PPAR-*γ* decreases IL-8 expression, thus inhibiting neutrophil migration^119^. In addition, binding of CBD to PPAR-*γ* decreases the expression of pro-inflammatory proteins such as TNF-*α*, IL-1*β*, and IL-6^14^. PPAR-*γ* has a putative binding site for CBD, and its activation by CBD induces not only anti-inflammatory effects but also promotes neurogenesis. Upon PPAR-*γ* activation, NF-*κ*B promoters are inhibited, leading to the marked downregulation of genes regulated by NF-*κ*B. Additionally, CBD-induced PPAR-*γ* activation has been shown to upregulate the expression of inducible nitric oxide synthase (iNOS) and cyclooxygenase-2 *(*COX-2), both of which are associated with neuroprotective effects^120^^,^^121^. CBD also interacts with members of TRP channel superfamily that are involved in sensing external stimuli such as temperature, mechanical pressure, and pH through neural signaling. Dysfunction in these channels is associated with conditions such as neuropathic pain, inflammation, and respiratory disorders^122^. Beyond sensory functions, TRP channels also play key roles in immune processes including cytokine production, phagocytosis, and cellular polarization, thereby contributing to the regulation of inflammatory responses^123^. Moreover, due to TRP channel expression in skin cells, topical application of CBD may influence pain and itch perception as well as support epidermal homeostasis^124^. Another potential mechanism by which CBD may mitigate fibrosis is through the reduction of ROS formation. In the study of Rajesh et al.^125^ on diabetic mice, CBD treatment increased the activity of SOD in the heart of diabetic murine, thus causing changes in both pro-survival and stress signaling pathways, as well as reducing fibrosis in diabetic myocardium by affecting NF-*κ*B activation. In a similar study Genovese et al.^126^ observed that while *SOD* gene expression increased, the expression of ROS-producing enzyme NOX decreased after CBD treatment in endometriosis-induced rats.

All the mentioned pathways demonstrate a molecular basis for CBD's anti-fibrotic properties, particularly through activation of PPAR-*γ* and the CB2 receptor. This outcome is supported by results from both *in vitro* and *in vivo* experimental models^34^. For example, a recent study demonstrated that in mice exposed to perfluorooctane sulfonate (PFOS)―a compound known to enhance macrophage-mediated inflammation and promote fibrosis―co-treatment with CBD modulated the coiled-coil domain containing 25 (CCDC25)―integrin-linked kinase (ILK)―NF-*κ*B signaling axis and effectively prevented liver fibrosis^127^. In another study, VCE-004.3, a CBD derivative, proven to be a dual antagonist to both PPAR*γ* and CB2 receptors and showed to inhibit TGF-*β*-mediated collagen synthesis myofibroblast differentiation. Furthermore, in the same study, VCE-004.3 prevented mast cell degranulation and macrophage infiltration in the skin, leading to reduced dermal thickness and decreased collagen accumulation around blood vessels in a murine model^128^. The same derivative was also used in an *ex vivo* analysis to evaluate ERK1/2 activation, a key event in myofibroblast differentiation, using immunoglobulin G (IgG) isolated from the peripheral venous blood of patients with systemic sclerosis or limited cutaneous systemic sclerosis. The study found that VCE-004.3 effectively inhibited ERK1/2 activation^129^ and this maybe the key point for prevention skin fibrosis. However controversial results also obtained for CBD. For example, Gurgul et al.^130^ found that low concentration of CBD reduced the apoptosis on ethanol-exposed human dermal fibroblasts however this effect was reversed at the higher concentration or the longer exposure time. In another recent study on methionine/choline deficient induced C57BL/6 male mice showed that high dose of CBG failed to reduce leukocyte infiltration, increase macrophage population and caused liver damage that can lead liver fibrosis^113^. These findings disclose the dual role of CBD on fibrosis which is mostly depended on factors such as dosage, timing, and the specific cellular environment. Therefore, understanding the effects of CBD on fibrotic processes is essential for its potential therapeutic application however further investigations are needed.

### 3.3 Muscle disorders

Muscle disorders such as muscular dystrophy and neuromuscular disorders impair muscle function by either directly damaging the muscular tissue and jeopardizing its regenerative capacity, or affecting the nerves that innervate them and are characterized by spam and pain^131^^,^^132^. Several studies have explored the effects of CBD on the symptoms of various muscle disorders and its potential in muscle damage, attenuation and regeneration. However, the method of CBD delivery plays a crucial role in determining its clinical efficacy, patient compliance, and safety profile.

Iannotti et al.^133^ investigated the potential use of CBD for the treatment of Duchenne muscular dystrophy (DMD)―a chronic inflammation and irreversible skeletal muscle degeneration. The research included measuring the effects of CBD (60 mg/kg) on the differentiation of murine myoblasts, primary satellite cells, and myoblasts isolated from healthy and DMD patients and also on locomotor activity in a murine model of DMD (male dystrophic mdx mice). The obtained results suggested that CBD stimulates the differentiation of murine and human DMD myoblasts and muscle satellite cells into myotubes. CBD also improved the impaired locomotor activity and muscle strength in MDX mice (a murine model of DMD), even at the late stages of the disorder, which is accompanied by a significant reduction in inflammation markers. Overall, the results showed that CBD has the potential to be used as a therapeutic agent for counteracting dystrophies. Nitecka-Buchta et al.^134^ evaluated the myorelaxant efficiency of CBD after the transdermal application in patients with myofascial pain (MFP), a condition associated with muscle damage and commonly affecting the masseter muscle. The study included 60 patients who were provided with a CBD-containing formulation for dermal application to the masseter muscle. The results showed that the application of CBD formulation reduced the activity of masseter muscles and improved the condition of masticatory muscles in patients with MFP137. This study clearly highlights the advantages of topical delivery, thereby avoiding hepatic first-pass metabolism and enabling more consistent therapeutic levels, which is especially beneficial in disorders requiring continuous symptom control.

Amyotrophic lateral sclerosis (ALS) is a severe and progressive neuromuscular disorder that results in the degeneration of the motor neurons in the cortex, brain stem and spinal cord. The first symptoms are characterized by progressive muscle weakness and loss of the ability to speak or swallow. Lacroix et al.^135^ conducted a large questionnaire-based survey about the “real-life” situation regarding cannabis use in the medical context in ALS patients in France. The most reported symptoms were muscle weakness, spasticity and rigidity. From 129 respondents, 28 reported using cannabis. Most of them were using CBD containing products (oil, herbal tea, weed and resin) and declared improvement in rigidity, muscle cramps, fasciculation, pain and sleep quality.

Myotonia is a genetic disorder that causes muscles to be unable to relax after they contract. Patients with dystrophic myotonia also have progressive muscle loss and weakness, as well as myalgia—muscle aches and pain. The symptomatic treatment of these patients is often not satisfactory. Some patients report symptom relief through the consumption of cannabis. In the study of Montagnese et al.^136^, six patients with dystrophic and non-dystrophic myotonia, to whom other therapies for myotonia and myalgia failed, were prescribed with CBD/THC oil as compassionate use. A low dose (mass ratio of CBD:THC = 10:1–10.29 mg CBD and 1.1 mg THC) was applied in the first two weeks and adjusted to a higher dose (mass ratio of CBD:THC = 6:1–20.58 mg CBD and 3.31 mg THC) in the following two weeks. The overall use was four weeks. All patients reported an improvement in myotonia, especially in weeks 3 and 4 of treatment. One of two patients with chronic myalgia experienced a significant improvement under treatment. Some gastrointestinal complaints, such as abdominal pain and diarrhea, improved in three patients; however, four out of six patients reported new-onset constipation. A more effective strategy may involve the use of controlled-release delivery systems, such as biodegradable nanoparticles or transdermal patches, offering an initial burst release for rapid symptom relief followed by sustained release. This approach could provide therapeutic plasma levels over extended periods, improving patient adherence and minimizing adverse effects related to peak concentrations. CBD nanoparticles, for instance, may be delivered either topically (provided adequate skin permeability) or *via* injection to target deeper muscle tissue. This strategy could significantly enhance local drug concentration and therapeutic action while avoiding systemic peaks that cause side effects.

Multiple sclerosis (MS) damages the CNS and some of the most common symptoms are spasticity, muscle spasms and neuropathic pain. CBD has shown in numerous trials to reduce stiffness, discomfort, inflammation and exhaustion in MS patients, resulting in increased mobility^137^. There are several research studies on nabiximols—an oromucosal spray containing a molecular ratio 1:1 of CBD and THC (trade name Sativex®) showing that they decreased spasticity and pain in MS patients in most trials^138^, ^139^, ^140^, ^141^. This oromucosal delivery route allows for rapid absorption *via* the buccal mucosa, bypassing the gastrointestinal tract and liver metabolism, and enabling fast symptom relief. This product is an example of well-optimized delivery system for cannabinoid-based therapies in neuromuscular conditions.

Overall, there is a lack of wider clinical trials on CBD use for muscle disorders and its adverse effect on muscle structure, nevertheless initial trials indicated its superior qualities in these conditions that could relieve the patient symptoms. In the future advanced delivery systems should be developed/improved and evaluated, like microneedle patches and encapsulated slow-release injectables.

### 3.4 Inflammation

Arthritis is a disease that is characterized by synovial inflammation, leading to joint damage and pain, as well as bone and cartilage destruction and deformation. The most common are osteoarthritis and rheumatoid arthritis (RA)^142^. Various research shows that CBD attenuates inflammation and pain^142^, ^143^, ^144^. However, its therapeutic efficiency is strongly influenced by the delivery method, which determines the absorption, bioavailability and consistency of the effects. Hammel et al.^143^ examined the efficacy of transdermal CBD application for reduction of inflammation and pain in a rat adjuvant-induced monoarthritic knee joint model. CBD is hydrophobic and has poor oral bioavailability, therefore topical application avoids gastrointestinal administration, first pass metabolism, and provides more constant plasma levels. In this study, CBD gels were applied for four days after arthritis induction. The results showed that transdermal CBD gel significantly reduced joint swelling, limb posture scores as a rating of spontaneous pain, immune cell infiltration and thickening of the synovial membrane in a dose-dependent manner (6.2 and 62 mg/day were the effective doses). Exploratory behavior was not altered by CBD, indicating a limited effect on higher brain function. These findings highlight the importance of optimized delivery routes in enhancing therapeutic benefits. In other study, Lowin et al.^144^ investigated the effect of CBD on intracellular calcium, cell viability and cytokine production in synovial fibroblasts that are major contributors to joint destruction in RA. Synovial tissue samples were isolated from 40 patients with long-standing RA. The research showed that CBD might suppress arthritis by targeting synovial fibroblasts under inflammatory conditions by increasing intracellular calcium levels and reducing cell viability and IL-6/IL-8/MMP-3 production of RA synovial fibroblasts. A survey on the efficiency of CBD application on arthritis symptoms^142^ showed that from a total 428 participants, there was an improvement in pain (83%), physical function (66%) and sleep quality (66%). Only 41% of all respondents reported at least one side effect, where 84% were considered mild, 14% moderate and 2% severe. The most commonly reported side effects were dry mouth and drowsiness, while less frequently reported effects included changes in appetite (increase or decrease), dry eyes, dizziness, headache, and digestive complaints. The heterogeneity in responses may partially reflect the differences in delivery methods—pointing to the need for standardized and optimized formulations to improve consistency in therapeutic outcomes.

Several clinical trials registered on Clinical Trials data base (https://clinicaltrials.gov/) have also been conducted on CBD use for arthritis treatment (Table 2). The results of Phase 2 trial No. NTC04611347 showed a significant improvement after two weeks of CBD-containing topical cream application in patient-reported pain and outcome measures without altering physical parameters—grip and pinch strength and thumb range of motion^145^. This finding supports earlier preclinical data and illustrates that topical application may achieve meaningful symptom relief while maintaining safety.

**Table 2:** Clinical trials with CBD regarding arthritis treatment.

Identification No. and design limitations	Condition	Number of participants	Intervention	CBD administration form	Trial location
NCT04911127	Rheumatoid arthritis	67	200 and 400 mg of CBD twice daily	Oral capsule	United States
Randomized double-blind placebo-controlled					
NCT04611347	Joint arthritis	40	6.2 mg/mL of CBD 2 times daily	Topical application	United States
Randomized - recruiting					
NCT03693833	Hand osteoarthritis, psoriatic arthritis	136	10 mg of CBD daily for 2 weeks, twice for weeks 3 and 4, three times for weeks 5–12	Oral capsule	Denmark
Randomized placebo-controlled with blinded outcome					
NCT04607603	Osteoarthritis	86	200 mg of CBD three times daily	Oral capsule	Austria
Randomised double-blind placebo-controlled					

Periodontitis is a chronic inflammatory disease involving soft and hard tissue destruction in the periodontal region. The main bacterial pathogens of periodontal diseases are gram-negative anaerobic species. Due to the presence of these pathogens pro-inflammatory cytokines like IL-1*β* and TNF-*α* are activated and produced. Napimoga et al.^146^ tested the effects of injected CBD (5 mg/kg, daily, for 30 days) in a periodontitis experimental rat model and explored possible mechanisms underlying these effects. The morphometrical analysis of alveolar bone loss demonstrated that CBD-treated animals presented a decreased alveolar bone loss. The gingival tissues showed decreased neutrophil migration (MPO assay) associated with lower production of IL-1*β* and TNF-*α*. Chen et al.^147^ also investigated the anti-inflammatory effects of CBD in periodontitis model in rats but through oral application of CBD-containing ointment (5 mg/kg, daily, for 28 days). The results showed that CBD attenuates periodontitis—the alveolar bone resorption was inhibited by reducing the secretion of TNF-*α* and IL-1*β*, indicating that both topical and injected CBD application is effective in treating periodontitis. These findings highlight that **local delivery** can be effective, but the choice of route may impact the treatment design and specificity. Furthermore, the only registered and completed clinical trial (randomised, double-blind and placebo-controlled), “CBD effect on periodontal health of patients with chronic periodontitis” (ClinicalTrials.gov identifier: NCT05498012), demonstrated a statistically significant improvement after CBD application. In a study involving 30 patients, treatment consisted of applying a local drug delivery—a dental gel containing 1% *w*/*w* CBD three times, followed by the use of a toothpaste containing 1% *w*/*w* of CBD for 56 days. No adverse effects of CBD were reported by the patients or observed during the study^148^.

Another chronic inflammatory disorder is psoriasis. It is characterized by hyperproliferation and dysregulated differentiation of keratinocytes. There are several studies where CBD has shown its potential in the treatment of psoriasis. In 2017 a phase I clinical trial (randomised, double-blind and placebo-controlled) to determine the safety and tolerability of topical cream containing 3% *w*/*w* CBD and 3% *w*/*w* THCwas sponsored by One World Cannabis Ltd (ClinicalTrials.gov identifier: NCT02976779). The results were not shared, but in 2020 a patent on a pharmaceutical topical composition comprising CBD and THCwith 1:1 molecular ratio was granted^149^. One clinical trial with five patients was conducted using a commercial CBD-containing ointment (Hemptouch Ltd., Novo Mesto, Slovenia) two times daily for three months. The ointment improved the skin condition and the Psoriasis Area Severity Index (PASI)^150^. The latest study of two topical CBD-containing creams (1.35 mg/g THC and 1.25 mg/g CBD without and with polyherbal formulation) showed a significant reduction in the psoriasis severity after four weeks, observed through the PASI score. This study was conducted involving 20 volunteers^151^. Although these results are promising, future studies should systematically explore the relationship between formulation composition, skin permeability, and clinical outcomes to guide the product development. Currently, no additional clinical studies are being conducted to explore the potential of CBD for the treatment of psoriasis. In summary, suboptimal outcomes may be achieved due to poor absorption and first-pass metabolism in oral formulations, while local transdermal and topical delivery can improve the bioavailability and clinical consistency.

**Figure 4:**
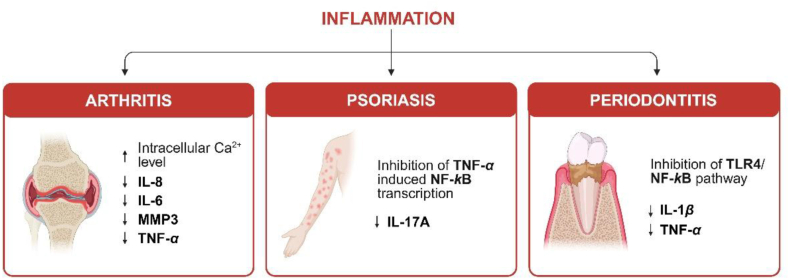
Anti-inflammatory activity of CBD in arthritis, psoriasis and periodontitis. Figure created with BioRender.

The anti-inflammatory activity of CBD in the treatment of arthritis, psoriasis and periodontitis is summarized in Fig. 4.

## 4 Strategies for local CBD delivery in soft tissue wound healing

**Figure 5:**
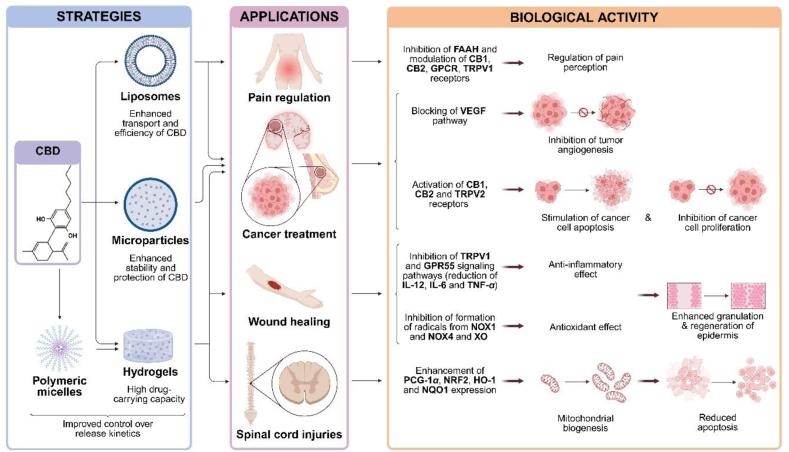
Strategies for delivery of CBD. Figure created with BioRender.

The unique therapeutic properties of CBD hold great promise for the treatment of different diseases and disorders, including alleviating pain associated with various soft tissue injuries or conditions, fibrosis, inflammation, and addressing muscle disorders. The most widely applied CBD delivery systems for soft tissue treatment are oral, topical and nasal. However, CBD application is limited due to low aqueous solubility^62^ and physicochemical stability issues associated with pH (the optimal stability is at pH range 4–6)^152^, temperature (degradation even when stored at 37 °C) and sensitivity to light^153^^,^^154^. To enhance CBD delivery into soft tissues and maximize its therapeutic efficiency, several strategies and advanced delivery systems are currently under investigation (Fig. 5).

**Figure 6:**
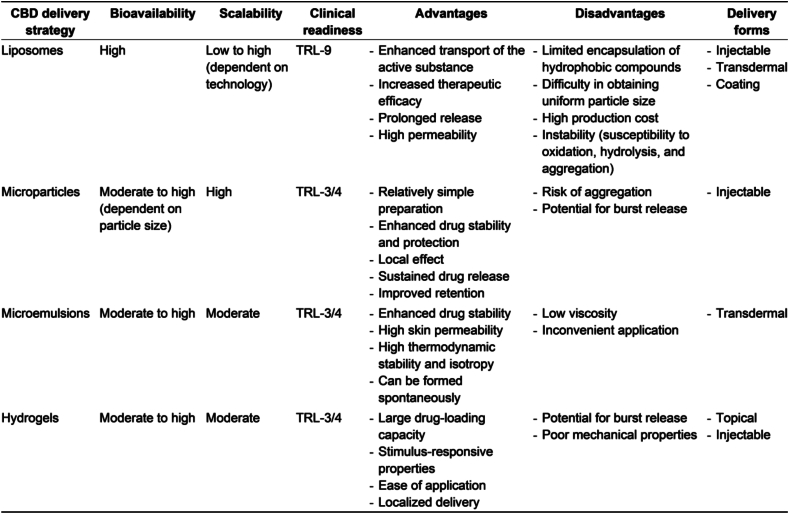
Comparative analysis of key indicators across CBD delivery strategies. (TRL—Technology Readiness Level, representing the highest available stage for each strategy)

Encapsulating CBD in various carrier systems enhances its physicochemical stability. For instance, lipid-based nanoparticles have been shown to improve both the thermal and photostability of CBD^155^, while alginate-based hydrogels contribute to enhanced thermal stability^156^. Additionally, a patented formulation involving CBD-loaded microparticles has demonstrated the ability to stabilize CBD oil, thereby improving its pharmacokinetic profile and bioavailability^157^. In this section, we overview the different strategies to encapsulate and deliver CBD. Furthermore, we have summarized and compared the bioavailability, scalability, clinical readiness, advantages, disadvantages and delivery forms of described CBD delivery strategy (see Fig. 6).

### 4.1 Liposomes

Liposomes are structurally like cell membranes and are formed by spherically arranged amphiphilic phospholipid molecules arranged in two layers when phospholipids are hydrated in an aqueous medium^158^. The main benefits of liposomes include enhanced transport and efficacy of biologically active substances. However, there are limitations in their ability to incorporate hydrophobic drugs as well as potential instability due to susceptibility to oxidation and hydrolysis^159^^,^^160^. Thus, obtaining CBD-loaded liposomes can be challenging due to its low aqueous solubility^61^. As a hydrophobic compound, CBD integrates into the bilayer of the liposome rather than the aqueous core.

Although lipid-based carriers and cannabis oils^161^ are commercially available in the market as CBD delivery systems (such products as Epidiolex®^162^^,^^163^) there is a notable lack of research studies and detailed descriptions of the preparation techniques for these systems. Liposomal preparations have been proposed by Valh et al.^164^ using the emulsification method. The formulation consisted of lecithin and sunflower oil, resulting in liposomes with an average size of 1900 nm and a 90% encapsulation efficiency for CBD (CBD used as an extract in oil). The obtained liposomes were used to coat sanitary materials, aiming to regulate pain and promote muscle relaxation during the menstrual cycle.

CBD encapsulation through film rehydration combined with probe ultrasonication was done by Fu et al.^165^ and the thin film hydration by Franzé et al^166^. With these methods, it was possible to obtain liposomes with particle sizes of 100–200 nm. However, in both studies CBD was encapsulated together with another bioactive agent. To ensure the anti-tumor properties of the obtained system, CBD was co-encapsulated with 20(*S*)-protopanaxadiol (PPD) in liposomes prepared from Soybean phosphatidylcholine, obtaining CBD-PPD liposomes (CP liposomes). The CP liposomes were used to determine *in vivo* anti-tumor efficiency in mice with breast cancer (4T1 cell line) tumors. To evaluate the synergetic effect of PPD and CBD, liposomes containing just PPD (15 mg/kg of PPD) and just CBD (15 mg/kg of CBD) were used. All liposomes were intravenously injected through the tail vein every 2 days, in total 7 times. CP liposomes were used in three concentrations-5, 15 and 45 mg/kg. The calculated tumor inhibition rate (TIR) demonstrated a dose-dependent anti-tumor efficiency, achieving TIR of 82% for CP liposomes with 45 mg/mg. At the same dose of 15 mg/kg, the TIR for liposomes containing just CBD and PPD (46.8% and 50.8%, respectively) was significantly lower than for CP liposomes (67.4%). These results demonstrated that the liposomal co-encapsulation of CBD and PPD can increase the anti-tumor efficiency, indicating on increased bioavailability. The *in vitro* CBD release data showed prolonged release that continued for up to 144 h^165^. Franze et al.^166^ co-encapsulated CBD with lidocaine in liposomes prepared from SPC to treat inflammatory-based skin disorders. The highest CBD encapsulation efficiency (92%–94%) was obtained when micellar dispersion of lidocaine was used during film rehydration instead of buffer with pH 2.5. The *in vitro* permeability studies were performed by using Franz diffusion cells and porcine ear skin as a membrane. The obtained skin permeation flux of CBD (0.19 ± 0.1 μg/cm^2^/h) was significantly higher than of 1% CBD solution in poly(ethylene glycol) 400 (0.03 ± 0.10 μg/cm^2^/h) that was used as a reference. In Jurgelane et al.^167^ research pure CBD was encapsulated in liposomes by using a thin film hydration method. The liposomes were prepared from various phospholipids. The incorporation of CBD reduced the size of all liposomes, achieving around 300 nm for liposomes prepared from distearoyl phosphatidylcholine (DSPC) and 1,2 distearoyl-*sn*-glycero-3 phosphoethanolamine-*N*-[carbonyl-amino(polyethylene glycol)-4300] (ammonium salt) (DSPE-PEG). These DSPC DSPE-PEG CBD liposomes also showed the highest encapsulation efficiency of 87% and a sustained CBD release, achieving 79% release over 504 h. *In vitro* viability tests with gingiva-derived mesenchymal stem cells (GMSC) showed that blank liposomes were non-cytotoxic. However, CBD-loaded liposomes significantly reduced cell viability for defined type of CBD containing liposomes. By comparing the obtained *in vitro* results with other *in vitro* studies of using pure CBD, it was concluded that the encapsulation of CBD in liposomes enhances its bioavailability, allowing lower CBD concentrations to be directly delivered to cells.

Research has been conducted also on using liposomes to deliver antibacterial agents to soft tissue. In such applications, antibacterial therapeutics can be encapsulated either inside the liposome core or embedded in the bilayer. The most frequently used antibacterial therapeutics include silver sulfadiazine^168^, amphotericin B^169^, azithromycin^170^ and vancomycin^171^. Despite this progress, there remains a significant gap in the literature regarding antimicrobial liposomes specifically designed for soft tissue applications. This highlights an opportunity for further investigation, particularly in light of existing studies demonstrating the antibacterial activity of CBD, as shown by Gildea et al.^172^ and Abichabki et al^173^.

### 4.2 Microparticles and microemulsions

Microparticles have gained attention as a drug delivery platform due to their ability to enhance drug stability and protection, as well as facilitate localized, targeted, or sustained release of drugs. Their unique properties are attributed to their distinctive form and structure, represented by their size within a diameter range of 1 to 1000 μm, consisting of a core surrounded by one or more membranes or shells^174^, ^175^, ^176^.

By regulating the microparticle surface properties, drugs or biomolecules can be conjugated outside the particle or encapsulated inside the particle^177^. A structure where there is no separation between the core and the membrane, with the drug dispersed in a polymer matrix, is termed a microsphere. On the other hand, a microcapsule refers to the core containing the drug surrounded by a shell. In this context, Perez de la Ossa et al.^178^ prepared CBD-loaded poly(epsilon-caprolactone) (PCL) microspheres for sustained CBD delivery. PCL was selected to mitigate the stability issues of CBD, which can arise from acidic conditions generated during the biodegradation of other aliphatic polyesters like poly(lactide) or poly(glycolide). In this study, the emulsion solvent evaporation technique, specifically oil-in-water were utilized to produce microspheres within a size range of 20–50 μm. As a result, the sustained release of CBD was observed after a single administration, with approximately 80% of CBD release achieved on Day 7. Additionally, the antitumoral effect *in vitro* was investigated using human breast cancer cell line (MDA-MB-231), revealing that all CBD microparticles inhibited proliferation of these cells for nine days^178^. The antitumoral effect of CBD is ensured by the induction of the apoptotic cancer cell death and inhibition of cancer cell proliferation due to the activation of CB1, CB2 and TRPV2 receptors. Additionally, it inhibits tumor angiogenesis by blocking vascular endothelial growth factor (VEGF) pathway^179^^,^^180^. In another work, the same research group^181^ studied PCL microparticles with an average size of ∼50 μm loaded with CBD and THC, prepared with the same evaporation technique. The sustained release was observed for both types of microparticles (containing CBD and containing THC) for 13 days. The potential anticancer effects of CBD, THC, and their combination encapsulated in microparticles were investigated in mice with induced human glioma, by peritumoral injection of microparticles every five days. To evaluate the efficiency of encapsulation of CBD and THC, solutions containing only CBD, only THC and a mixture of both were also administrated to these mice, but with a daily peritumoral injection. The cannabinoid (CBD and/or THC) solutions were prepared in PBS with 1% *v*/*v* dimethyl sulfoxide and 5 mg/mL of bovine serum albumin, containing equivalent amount of both cannabinoids. The administration of the prepared cannabinoid microparticles every five days reduced the tumor growth with the same efficiency than the daily administration of cannabinoid solutions. Nevertheless, 59% of the initial CBD amount encapsulated in the microparticles were still present in the remaining microparticles at the site of injection at the end of the experiment. These results suggest that CBD and THC loaded microparticles could be used as an effective method in anticancer therapies and the necessary concentrations of CBD and THC could be reached at the tumor site by injecting less frequently. Fraguas-Sánchez et al.^182^ investigated PCL microparticles loaded with CBD in a mouse model to characterize *in vivo* release of CBD. These microparticles, with a size range not exceeding 60 μm and an encapsulation efficiency of up to 100%, were administered *via* injection. The study demonstrated that the CBD-loaded microparticles achieved continuous release following a single subcutaneous injection.

Another approach for delivering lipophilic compounds such as CBD to soft tissue is using microemulsions, which are defined as stable isotropic dispersions consisting of water, oil and amphiphilic compounds^183^^,^^184^. For example, a CBD-loaded microemulsion was investigated for enhanced stability and transdermal delivery. Park et al.^185^ developed a CBD-loaded microemulsion based on Capryol® 90 as an oil phase and Procetyl™ AWS as a surfactant, along with ethanol and distilled water. Remarkably, the microemulsion, comprising 95% *w*/*v* CBD, remained stable for 180 days. However, microemulsions are generally characterized by low viscosity, which could be a drawback in developing some systems for topical drug delivery due to the inconvenient application to the skin^186^^,^^187^. In this context, the local delivery of CBD to soft tissue was also considered through microemulgel formulations, representing CBD-loaded microemulsion in a microgel form^188^. This microemulsion included an oil phase-isopropyl myristate, Solutol® HS 15 as a surfactant, Transcutol® P as a cosolvent and water. The microemulgel (MEgel) was obtained by jellifying the microemulsion with a thickening agent and hydrogel Sepigel® 305 (CBD-gel). The comparison between CBD-loaded MEgel and solely CBD-gel showed stability in both formulations over three months. Furthermore, the CBD release *in vitro* was conducted for 24 h using the Franz diffusion cell system with 5% *v*/*v* Tween 20 in PBS solution as the release medium. The release from CBD-gel was slower but more uniform than from CBD-loaded MEgel. However, after 24 h the CBD-gel had released more CBD (128 ± 20 μg/cm^2^) than CBD-loaded MEgel (90 ± 24 μg/cm^2^). The results of transdermal permeation through rabbit ear skin *ex vivo* showed that the CBD amount adsorbed and retained inside the skin after 24 h was significantly higher for CBD-gel (11 ± 5 μg/cm^2^) than for CBD-loaded MEgel (5.3 ± 1.4 μg/cm^2^). The CBD-loaded microemulsion gel (MEgel) demonstrated slower and more controlled CBD diffusion, with minimal transdermal absorption compared to a conventional CBD gel, making it more suitable as a dermal delivery system for topical application^188^. Overall, this and other previously mentioned studies underscore the potential of microparticulate systems for delivering CBD and other lipophilic cannabinoids to soft tissues effectively.

### 4.3 Hydrogels

Hydrogels possess a high drug-carrying capacity, making them versatile for various treatments by enabling the loading of different active substances, drugs, or cells^189^^,^^190^. A novel CBD oil containing chitosan-based hydrogel (CBD-CS) has been reported as a potential biomaterial for wound healing. CBD-CS exhibited good antimicrobial activity. Samples with 5% *w*/*w* of CBD rapidly released 13% of the initial CBD in the first 20 min, suggesting that this sample could be potentially used as a drug carrier in rapid drug release applications. After 85 h this sample (5% *w*/*w* of CBD) reached the highest CBD release (19.4%). In addition, cell culture studies demonstrated that CBD-CS is biocompatible^19^. Other CBD-loaded hydrogel dressing made of alginate crosslinked with zinc ions (CBD/Alg@Zn) for wound healing were designed by Zheng et al^191^. In the first 25 h, a fast CBD release was observed (approximately 50%‒70%, depending on the initial CBD concentration) and then again, a significantly slower release rate was observed. *In vivo* studies in rats indicated that CBD/Alg@Zn exhibited an excellent wound healing effect by showing more granulation tissue, regenerated epidermis and milder inflammatory cell infiltration after seven days comparing to control, commercial wound dressing and hydrogel Alg@Zn. Granulation tissue formation and regeneration of epidermis are promoted by the antioxidant and anti-inflammatory effects of CBD. It prevents the radical formation from NOX1, NOX4 and xanthine oxidase (XO), and inhibits TRPV1 and G protein-coupled receptor 55 (GPR55) signaling, thereby reducing levels of IL-12, IL-6 and TNF-*α*. Also, a relatively large number of blood vessels and some hair follicle structures were observed in the CBD/Alg@Zn group on Day 14. Whereas Zhang et al.^190^ developed an injectable *in situ* gelling hydrogel of chitosan (CS) and sodium carboxymethylcellulose loaded with 2% *w*/*w* CBD for treating spinal cord injury (SCI) in a rat model. The CBD release was ∼40 % in the first 8 h, and then it gradually slowed down, reaching slightly over 60% after 72 h, and resulting in reduced apoptosis in SCI by enhancing expression of peroxisome proliferator-activated receptor-*γ* coactivator (PCG-1*α*), nuclear factor erythroid 2-related factor 2 (NRF2), heme-oxygenase 1 (HO-1) and NAD(P)H quinone dehydrogenase 1 (NQO1) and therefore mitochondrial biogenesis^190^. Finally, CBD can be loaded into the hydrogels indirectly—*via* polymeric micelles. Kamenova et al.^192^ developed an injectable *in situ* gelling hydrogel containing CBD-loaded polymeric micelles. Basically, all encapsulated CBD was released gradually over a period of 10 and 12 h for samples containing 0.1 and 0.5 g/L of CBD, respectively. The release occurred simultaneously with the erosion of the hydrogel. The CBD-loaded polymeric micelles were non-cytotoxic but the injectable hydrogel containing these micelles showed pronounced activity against malignant MCF-7 human tumor cells. Combining microparticles and/or nanoparticles with monolithic delivery systems represents a promising approach for achieving improved control over drug release kinetics. In conclusion, these studies emphasize the potential of hydrogels as effective drug delivery systems for CBD delivery to soft tissues and their application in wound healing, highlighting their promising future in clinical settings.

## 5 Challenges and perspectives

**Figure 7:**
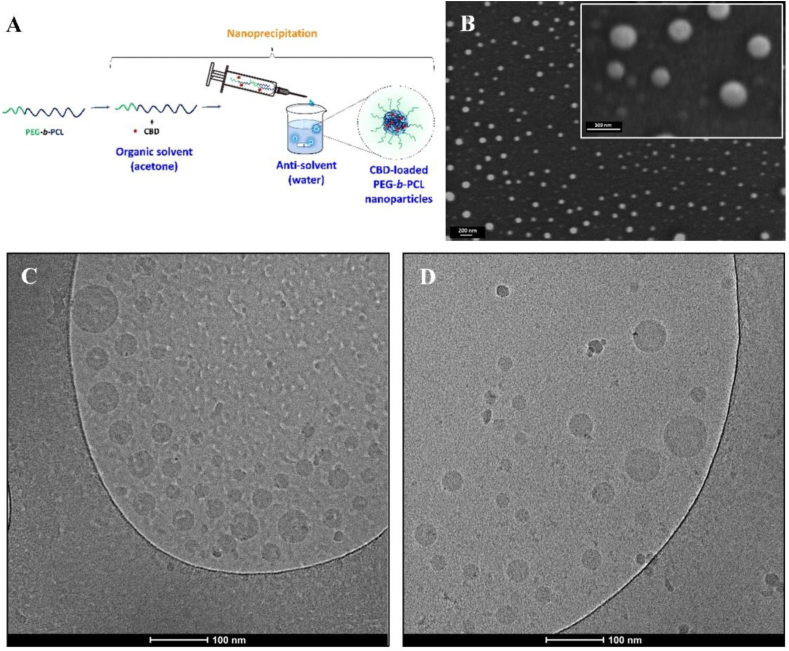
Synthesis and characterization of CBD-loaded PEG-*b*-PCL nanoparticles. (A) Schematic representation of the synthetic method. (B) Representative HR-SEM micrograph of CBD-free and CBD-loaded nanoparticles. (C, D) Representative HR-cryo-TEM micrograph of (C) CBD-loaded nanoparticles and (D) CBD-loaded nanoparticles with 4.5% *w*/*v* of 2-hydroxy-propyl-beta-cyclodextrin in the suspension. The CBD loading (%DL) in all the nanoparticles was 11% *w*/*w* and the nanoparticle concentration was 0.1% *w*/*v*. Reproduced with the permission from Ref. 196. Copyright ® 2023 Springer Nature Link.

**Figure 8:**
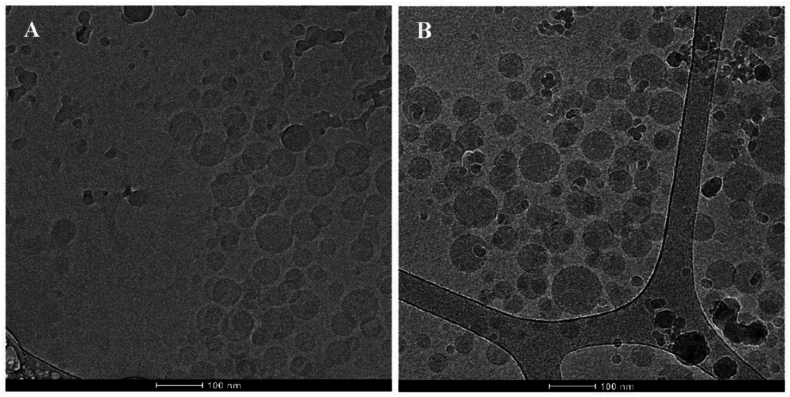
Representative cryo-TEM micrographs of fresh (A) CBD-loaded non-crosslinked mixed chitosan-*g*-poly(methyl methacrylate)/poly(vinyl alcohol)-*g*-poly(methyl methacrylate) polymeric micelles and (B) CBD-loaded sodium tripolyphosphate-crosslinked mixed chitosan-*g*-poly(methyl methacrylate)/poly(vinyl alcohol)-*g*-poly(methyl methacrylate) polymeric micelles. The CBD loading in the polymeric micelles was 20% *w*/*w*. Reproduced from Ref. 197 Open access Creative Common CC BY license.

The historical use of cannabinoids in ancient medicine, coupled with modern preclinical investigations into their various components, unveils a broad spectrum of its potential clinical applications for soft tissue regeneration. Despite its promise, significant challenges remain. There is scientific evidence on the benefit of cannabis extracts over the pure cannabinoids owing to the adjuvant and synergic role of terpenes. A major limitation in the use of extracts is the difficulty of ensuring their reproducible composition among plant strains, batches and producers. From the point of view of pharmacological research, formulation development and regulatory approval, the use of the individual cannabinoids is advantageous due to the ability to constrain its pharmacological effect, investigate the pathways involved and link a certain effect to a specific compound, and the possibility to rationally develop formulations and delivery systems, and administration strategies that address the biopharmaceutical drawbacks of each one of them. In the case of CBD, recent breakthroughs have overcome its poor aqueous solubility and low chemical stability by encapsulating it within different types of molecular carriers (*e.g*., cyclodextrins)^193^, particulate systems including nanoparticles^194^ and incorporating the loaded particles into hydrogels that can be administered locally in wounds^195^. For example, CBD has been incorporated into liposomal bilayers^164^ or nanoparticles made from hydrophobic polymers like PCL through nanoprecipitation to increase its oral bioavailability with respect to free CBD in rat (Fig. 7)^196^ or into mixed self-assembled amphiphilic polymeric nanoparticles made of mucoadhesive chitosan and poly(vinyl alcohol) that showed good permeability across different biological barriers such as a model of the corneal epithelium *in vitro* (Fig. 8)^197^.

These strategies are currently combined with alternative administration routes that ensure localized treatment. Additionally, integrating CBD-loaded microparticles and nanoparticles with injectable hydrogels that can be implanted using minimally invasive routes represents a modular and versatile approach for achieving better control over the spatiotemporal release kinetics of CBD in the body site of interest.

The potential of these approaches for soft tissue applications is promising; however, many studies are limited to *in vitro* research, while others lack comprehensive *in vivo* investigations. In future research, it is essential to investigate how the encapsulation affects the intrinsic physicochemical properties of CBD, such as its thermal and photostability. While initial studies suggest that certain carriers, like lipid nanoparticles^155^ and hydrogels^156^, can enhance CBD's stability, a more systematic evaluation across different encapsulation materials and conditions is needed. Understanding these effects will be crucial for optimizing formulation strategies and ensuring consistent therapeutic performance, especially in products exposed to variable environmental conditions during storage or use. Extensive studies on the efficacy and safety of CBD-loaded formulations are essential before they can progress to clinical trial approval. Consequently, there is currently a limited number of clinical trials focusing on these methods of CBD administration for soft tissue treatment.

In 2018, the FDA approved the CBD formulation known as Epidiolex® (Greenwich Biosciences, Carlsbad, USA), based on cannabis oil, acknowledged for its proven efficacy in treating severe forms of epilepsy^163^, sparking a variety of clinical trials. These trials are actively exploring the effect of CBD on patients with diverse health issues, thereby reinforcing its legitimacy as a promising therapeutic agent^198^. Completed clinical trials, evaluating the effects of CBD primarily focused on pain relief, severe epilepsy, and drug-resistant seizures syndromes, including Sturge-Weber, Lennox-Gastaut, Dravet and Tuberous Sclerosis Complex with CBD administered orally^38^. These trials demonstrated promising results that were related to the improvements in cognitive abilities, motor functions, and a reduction in seizure frequency^199^, ^200^, ^201^. Currently, commercially available products include Marinol® (AbbVie Inc., North Chicago, USA) and Syndros® (Benuvia Therapeutics, Round Rock, USA), both based on dronabinol (a synthetic THC), as well as Cesamet® (Meda Pharmaceuticals Inc., Canonsburg, USA), which contains the synthetic cannabinoid nabilone. Moreover, oromucosal spray Sativex® (GW Pharma Ltd.), based on nabiximols (a cannabis extract), is available for treating spasticity in multiple sclerosis is commercially available products involving CBD in soft tissue. It combines equimolar concentrations of THC and CBD^52^. Ongoing trials that are yet to yield results explore diverse routes of CBD administration, such as incorporating CBD into a dental gel to achieve periodontal maintenance effects^202^ and investigating its hydration properties and skin penetration abilities^203^. Additional studies delved into skin-related disorders like atopic dermatitis and acne, administering CBD in liquid formulation applied topically. However, these trials did not demonstrate significant success in treatment or improvement in patients’ conditions, notably, when used externally, patients experienced a similar number of adverse events as the placebo groups^204^^,^^205^. These findings emphasize the therapeutic potential of CBD in managing a variety of soft tissue-related conditions, indicating the need for ongoing research to enhance and refine its clinical applications. Multiple ongoing trials are currently investigating various conditions, signifying an expanding and diverse research landscape that remains undiscovered in the soft tissue recovery field. At the same time, it is worth mentioning that they do not necessarily utilize pure CBD as the active pharmaceutical ingredient and contain different cannabinoid combinations. In this regard, the clinical potential of CBD remains to be demonstrated, especially in the treatment of soft tissue lesions and regeneration.

## Author contributions

Arita Dubnika: conceptualization, writing – original draft, review & editing, supervision, resources, project administration, funding acquisition; Inga Jurgelane: writing – original draft, review & editing; Andra Grava: writing – original draft; Selay Tornaci: writing – original draft, review & editing, visualization; Natalia N. Porfiryeva: writing – original draft; Diana Solovyov: writing – original draft; Nabanita Saha: writing – original draft, review & editing, resources; Nibedita Saha: writing – original draft, review & editing; Elina Kelle: writing – review & editing, visualization; Dagnija Loca: writing – review & editing, conceptualization; Ebru Toksoy Öner: writing – review & editing, conceptualization, supervision, resources; Alejandro Sosnik: writing – original draft, review & editing, validation, supervision, resources.

## Declaration of Generative AI and AI-assisted technologies in the writing process

During the preparation of this work the authors used Generative AI tool ChatGPT GPT-4 foundation model in order to revise wording and to improve the writing style of this review article. After using this tool, the authors reviewed and edited the content as needed and take full responsibility for the content of the published article.

## Conflicts of interest

The authors declare no conflicts of interest.
